# Juvenile idiopathic arthritis treated with biological therapies

**DOI:** 10.1186/1479-5876-10-S3-P46

**Published:** 2012-11-28

**Authors:** Maria Luisa Velloso Feijoo, Rosalia Martinez Perez, Julia Uceda Montañes, Jose Luis Marenco de la Fuente

**Affiliations:** 1 Rheumatology Unit, Valme University Hospital, Seville, Spain

## Background

Biological therapies have dramatically changed the prognosis for children with juvenile idiopathic arthritis (JIA). There are doubts about the possibility of discontinuing treatment once remission is achieved. We focus in this question in our series.

## Objective

To assess the efficacy and safety of these drugs in our series of patients with JIA.

## Materials and methods

We identified 9 children with JIA treated with biologic therapies, and we made a description of our experience.

## Results

The mean age was 14.55 ± 5.85, with a female predominance (66.7%). At diagnosis, mean age was 4.94 ± 2.9, and at the beginning of biological treatment of 8.77 ± 2.63. The median time from diagnosis to initiation of biological therapy was 3.94 ± 2.83 years. The disease characteristics are detailed in the table.

**Table 1:** 

Patient	Sex	Age	Type	RF	ANA	Uveitis	Swollen joints
1	Female	10	Systemic	Negative	Positive	No	Knee
2	Female	13	Polyarticular	Negative	Negative	No	Knee and wrists
3	Male	10	Systemic	Positive	Positive	No	Knee and wrists
4	Female	10	Polyarticular	Negative	Negative	No	Temporomandibular and wrists
5	Female	11	Polyarticular	Negative	Negative	No	Wrists and metatarsophalangeal
6	Male	7	Oligoarticular	Negative	Negative	No	Ankles
7	Male	7	Oligoarticular	Negative	Negative	No	Knees
8	Female	5	Oligoarticular	Negative	Positive	Yes	Knees
9	Female	6	Oligoarticular	Negative	Negative	No	Ankles

All the children had previously received DMARDs (66.6% methotrexate (MTX) and 33.3% MTX and sulfasalazine). Eight of the 9 patients (88.9%) were taking corticosteroids at baseline. Eight received etanercept (ETN) and one Adalimumab (ADA), with good outcomes in all the patients unless 1 that had to switch from ETN to ADA due to inefficacy, and improved after the change. The steroids were suspended in 75% of children (6). Differences between mean values of CRP, ESR, and platelets from baseline to actual moment were statistically significant.

The median biologic time is 4 (1.11) years.

Actually all the children are in remission, two of them (patients 1 and 4) without biological treatment or classic DMARDs (since 5 and 2 years respectively).

None of the children have had significant adverse effects nor required hospitalization from the beginning of therapy.

## Discussion

ETN has proved its efficacy in JIA (regardless of the type of onset), as it has been reported in multiple efficacy and safety studies, including long term studies of up to eight years of continuous therapy.

We present our experience in children treated with up to 11 years, with good outcomes in terms of efficacy and safety in all the patients, and also 2 patients still in remission after 2 and 5 years without treatment.

## References

[B1] Giannini EH Long-term safety and effectiveness of etanercept in children with juvenile idiopathic selected categories of arthritis Arthritis Rheum 2009 60 9 2794 2804 10.1002/art.24777 19714630

[B2] Pratsidou-Gertsi P Trachana M Pardalos G Kanakoudi-Tsakalidou F A follow-up study of juvenile idiopathic arthritis Patients with etanercept discontinued due to Who disease remission Clin Exp Rheumatol 2010 28 6 919 22 Epub 2011 Jan 4 21205467

